# Proteostatic tuning underpins the evolution of novel multicellular traits

**DOI:** 10.1126/sciadv.adn2706

**Published:** 2024-03-08

**Authors:** Kristopher Montrose, Dung T. Lac, Anthony J. Burnetti, Kai Tong, G. Ozan Bozdag, Mikaela Hukkanen, William C. Ratcliff, Juha Saarikangas

**Affiliations:** ^1^ Helsinki Institute of Life Science, HiLIFE, University of Helsinki, Helsinki, Finland.; ^2^ Faculty of Biological and Environmental Sciences, University of Helsinki, Helsinki, Finland.; ^3^ School of Biological Sciences, Georgia Institute of Technology, Atlanta, GA, USA.; ^4^ Interdisciplinary Graduate Program in Quantitative Biosciences (QBioS), Georgia Institute of Technology, Atlanta, GA, USA.

## Abstract

The evolution of multicellularity paved the way for the origin of complex life on Earth, but little is known about the mechanistic basis of early multicellular evolution. Here, we examine the molecular basis of multicellular adaptation in the multicellularity long-term evolution experiment (MuLTEE). We demonstrate that cellular elongation, a key adaptation underpinning increased biophysical toughness and organismal size, is convergently driven by down-regulation of the chaperone Hsp90. Mechanistically, Hsp90-mediated morphogenesis operates by destabilizing the cyclin-dependent kinase Cdc28, resulting in delayed mitosis and prolonged polarized growth. Reinstatement of Hsp90 or Cdc28 expression resulted in shortened cells that formed smaller groups with reduced multicellular fitness. Together, our results show how ancient protein folding systems can be tuned to drive rapid evolution at a new level of biological individuality by revealing novel developmental phenotypes.

## Teaser

Down-regulation of Hsp90 decouples cell cycle progression and growth to drive the evolution of macroscopic multicellularity.

## INTRODUCTION

The evolution of multicellular organisms from single-celled ancestors has independently occurred ~50 times across the tree of life (*1*–*5*). Each of these events represents a major transition in individuality, but because they occurred in the deep past, relatively, little information is available about the evolutionary dynamics and molecular mechanisms through which simple groups of cells evolve into multicellular organisms.

The transition to multicellularity may precipitate a period of rapid evolution, as cells adapt to novel organismal and ecological contexts (*6*, *7*). Epigenetic mechanisms may play a crucial role in this process (*8*), as they are often capable of generating heritable phenotypic diversity at faster rates than mutation alone (*9*–*12*). In addition to mechanisms altering gene expression, many proteins exist in dynamic interconverting states of folding and assembly (*13*–*15*), which, in some cases, can produce heritable phenotypic variation that may serve as a basis for adaptive evolution (*16*, *17*). However, given the ancient origins of extant multicellular clades, no work has directly examined the role of epigenetic inheritance in the initial stages of the transition to multicellularity. Our experiment aims to circumvent this constraint through long-term directed evolution, providing insights into the potential role of nongenetic mechanisms during the early stages of this evolutionary transition.

Using long-term experimental evolution to select for larger size over thousands of generations, we recently showed that multicellular “snowflake yeast” can evolve to form multicellular groups that are more than 20,000 times larger and 10,000 times more mechanically tough than their ancestors (*18*). Cellular elongation played a central role in the evolution of these novel multicellular traits, allowing branches of cells to entangle with one another and thereby become orders of magnitude more mechanically tough (*19*). Here, we set out to investigate the underlying molecular mechanisms behind cellular elongation and macroscopic multicellularity. We found that down-regulation of the chaperone protein Hsp90, a key modulator of genotype-phenotype relationships, was a convergent adaptation underpinning the evolution of larger, more mechanically tough groups. Mechanistically, we found that reduced Hsp90 expression leads to modulation of cell morphogenesis by destabilizing the cyclin-dependent protein kinase Cdc28, resulting in delayed cell cycle progression and consequent cellular elongation. Collectively, these data unravel that altered chaperoning of cellular proteome can facilitate major evolutionary transitions by generating novel cell-level phenotypic traits that promote multicellular evolution.

## RESULTS

### Hsp90 is down-regulated during the evolution of macroscopic multicellularity

**Fig. 1.**
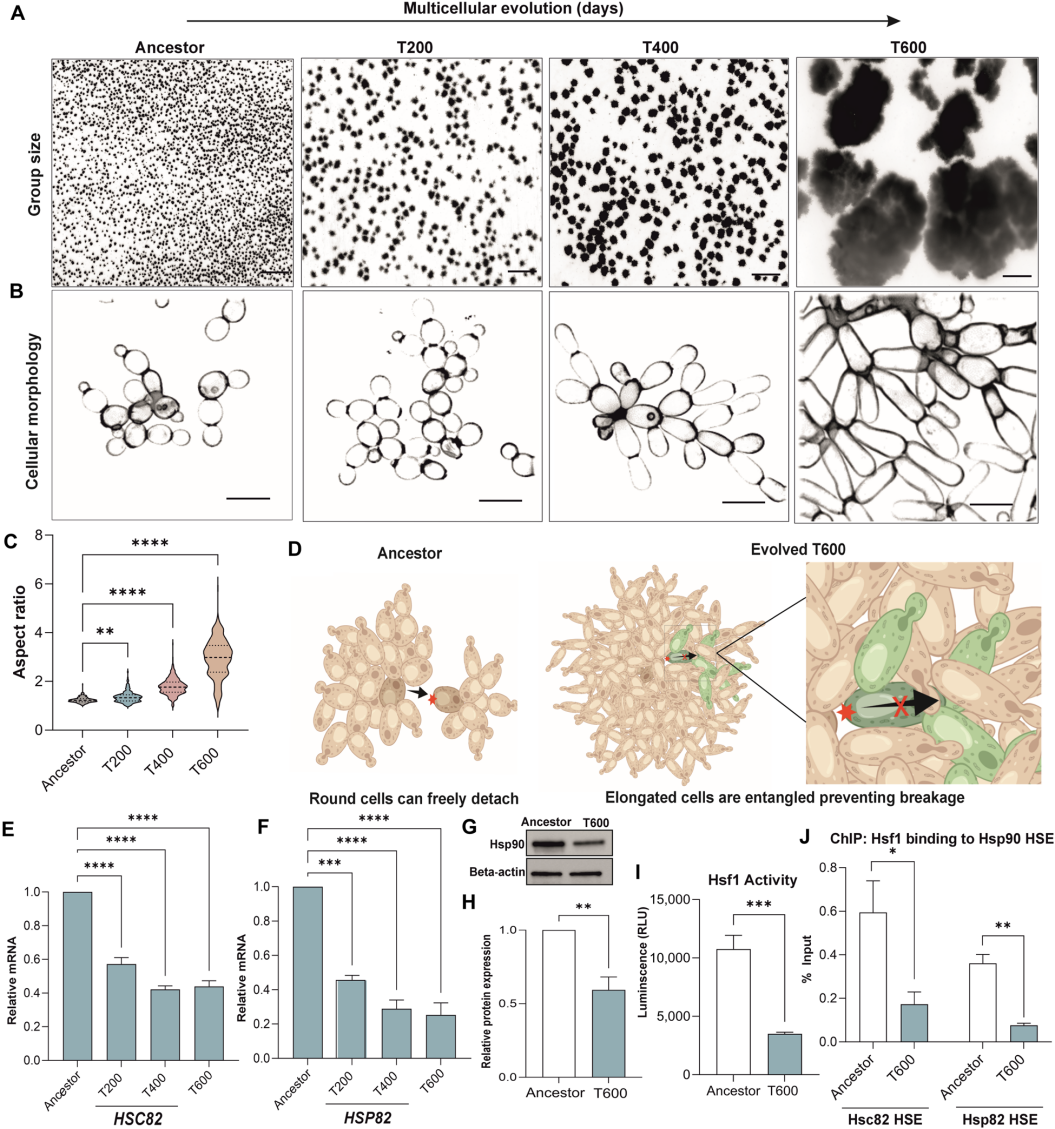
Hsp90 is down-regulated during the evolution of macroscopic multicellularity in snowflake yeast. (**A**) Representative images of snowflake yeast clusters from the MuLTEE experiment selecting for larger group size. (**B**) Representative images of calcofluor-stained snowflake yeast highlighting cell morphology during the MuLTEE. (**C**) Cellular aspect ratio quantification at different time points of the MuLTEE [*n* = 300 cells, *F*_3__,__1196_ = 220.5, *P* < 0.0001, one-way analysis of variance (ANOVA), Tukey’s post hoc test: Ancestor versus T200 *P* = 0.004, Ancestor versus T400 *P* < 0.0001, Ancestor versus T600 *P* < 0.0001]. (**D**) Graphic illustrating the effect cellular elongation has on entanglement and cluster robustness (*19*, *21*). Created with Biorender.com. (**E**) RT-qPCR quantification of *HSC82* expression in T200, T400, and T600 compared to Ancestor (*n* = 3, *F*_3__,__8_ = 96.65, *P* < 0.0001, one-way ANOVA, Tukey’s post hoc test: Ancestor versus T200 *P* < 0.0001, Ancestor versus T400 *P* < 0.0001, Ancestor versus T600 *P* < 0.0001). (**F**) RT-qPCR quantification of *HSP82* expression in T200, T400, and T600 compared to Ancestor (*n* = 3, *F*_3__,__8_ = 43.16, *P* < 0.0001, Tukey’s post hoc test: Ancestor versus T200 *P* < 0.0001, Ancestor versus T400 *P* < 0.0001, Ancestor versus T600 *P* < 0.0001). (**G**) Representative immunoblot of Hsp90 (Hsp82 and Hsc82) in Ancestor and T600. Beta-actin serves as a loading control. (**H**) Quantification of T600 Hsp90 relative band intensity compared to Ancestor Hsp90 (*n* = 3, *t* = 4.6, *P* = 0.0098, two-sample *t* test). (**I**) Hsf1 activity in Ancestor and T600 measured as luminescence (*n* = 4, *t* = 6.1, *P* < 0.0009, two-sample *t* test). (**J**) Hsf1 ChIP-qPCR analysis of Hsc82 and Hsp82 HSE regions in Ancestor and T600 (*n* = 4, Hsc82 *t* = 2.7, *P* = 0.026, Hsp82 *t* = 5.8, *P* = 0.002, two-sample *t* test). All values represent means ± SEM. Scale bars, (A) 500 μm and (B) 10 μm.

Our ongoing multicellularity long-term evolution experiment (MuLTEE) allows us to examine multicellular evolution in a nascent lineage of clonally developing organisms. This experiment was initiated in *Saccharomyces cerevisiae* strains lacking the *ACE2* open reading frame to generate small “snowflake” cell clusters (*20*, *21*) referred to hereafter as “Ancestors.” During the initial 600 rounds of size-based selection (~3000 generations) in aerobic, mixotrophic, and anaerobic snowflake yeast populations, only the anaerobic lines evolved macroscopic size (Fig. 1A) (*18*). This multicellular adaptation was the result of a morphological transformation from oval- to rod-shaped cells, leading to a significant increase in cellular aspect ratio (ratio of length to width) (Fig. 1, B and C). Elongated cells form long branches that mutually entangle, resulting in the evolution of far tougher multicellular groups (i.e., from weaker than gelatin to as strong as wood), allowing individual snowflake yeast clusters to grow to macroscopic size (Fig. 1D) (*18*).

To identify the molecular changes that underlie the morphological transformation from oval-shaped Ancestor cells to rod-shaped T600 cells, we examined the transcriptomes of anaerobic line 5 (PA5), which grew the largest clusters after 600 days of settling selection. Our results identified ~540 down-regulated genes and ~460 up-regulated genes (*P* < 0.05, log_2_ fold change cutoff 0.5) in the T600 cells compared to Ancestors. Examining the top 50 Gene Ontology (GO) terms revealed that the major differentially expressed genes were involved in metabolism, cell cycle, and translation (fig. S1A). We found that a number of genes encoding chaperone proteins were down-regulated in T600 cells including both isoforms of yeast Hsp90, *HSC82*, and *HSP82* (fig. S1B). Hsp90 is particularly interesting as it is involved in the final folding steps of specialized client proteins that include transcription factors and kinases and thereby controls their activity posttranslationally. Thus, it can modify the genotype-phenotype relationship by altering the activity of key developmental pathways (*22*, *23*).

We confirmed the RNA sequencing (RNA-seq) results using quantitative polymerase chain reaction (qPCR), which demonstrated that *HSC82* mRNA was reduced by ~2-fold and *HSP82* mRNA by ~3-fold in T600 cells as compared to the Ancestor (Fig. 1, E and F). Further analysis revealed that a decline in Hsp90 mRNA expression can be already observed starting from day 200 of evolution. Consistent with declining mRNA expression, Western blot analysis using an antibody that recognizes both Hsp90 isoforms revealed that Hsp90 declined by ~40% in T600 cells, relative to the Ancestor (Fig. 1, G and H).

To determine the cause of the Hsp90 decline, we investigated the activity of transcription factor Hsf1, which is responsible for the expression of yeast Hsp90 (*24*). Hsf1 binds to heat shock elements (HSEs) found in the promoter region of its target genes, such as Hsp90, to activate transcription. In yeast, Hsf1 is active at basal levels promoting transcription of constitutively expressed Hsc82 and it can be further induced by stress, such as heat, to promote the expression of stress-induced Hsp82. We measured the activity of Hsf1 by quantifying the expression of a genomically integrated luciferase reporter gene that is under the control of HSE promoter regions (*25*). Using luminescence output as a measure of Hsf1 activity, we found that there was less Hsf1 activity occurring in T600 cells compared to Ancestor cells at both the basal level (Fig. 1I) and after heat shock (fig. S1C). Our whole-genome sequencing detected no mutations in *HSF1*, the HSE regions of *HSP82* or *HSC82*, or in other regulators of the heat shock response. In addition, there were no differences in the expression of *HSF1* between the Ancestor and T600 cells (fig. S1D). To test whether the altered Hsf1 activity is mediated by reduced target gene binding, we used a chromatin immunoprecipitation (ChIP) assay to analyze the binding of Hsf1 to Hsp90 HSE promoter regions. Compared to the Ancestor, we found a ~70% reduction in Hsf1 occupancy at Hsp90 HSE regions in T600 (Fig. 1J), explaining the loss of activity and reduced Hsp90 expression. Together, these results indicate that Hsp90 down-regulation is mediated by its reduced transcription by Hsf1.

### Down-regulation of Hsp90 plays a critical role in snowflake yeast evolution

**Fig. 2.**
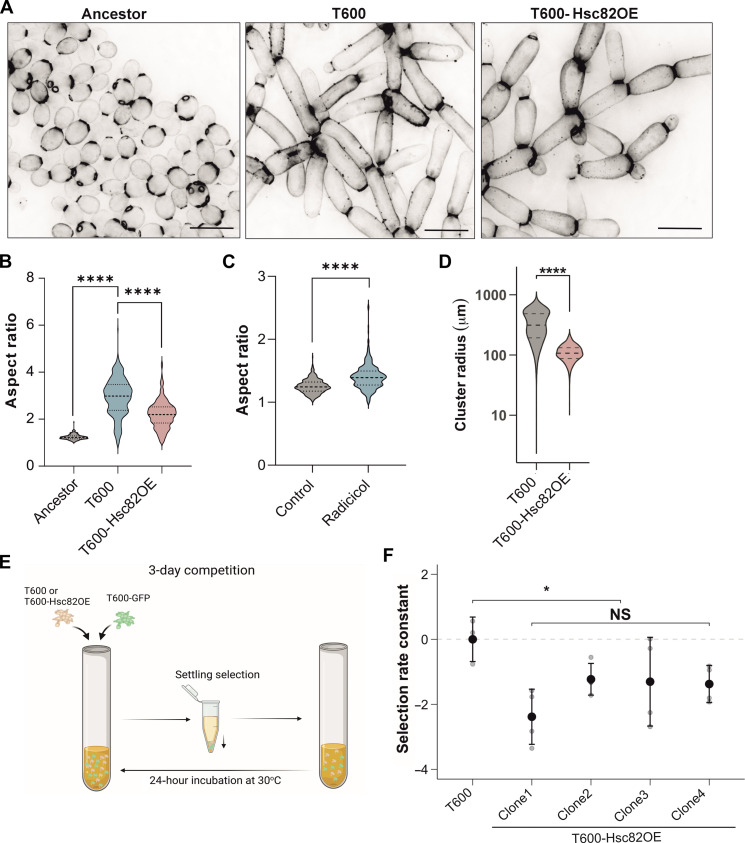
Overexpression of *HSC82* decreases the aspect ratio and cluster size of T600 cells. (**A**) Representative images of calcofluor-stained Ancestor, T600, and T600-Hsc82OE cells, highlighting differences in cellular morphology. Scale bars, 10 μm. (**B**) Cellular aspect ratio quantification of Ancestor, T600, and four T600-Hsc82OE clones combined (*n* = 300 cells, T600-Hsc82OE, *F*_2,897_ = 665.9, *P* < 0.0001, one-way ANOVA, Tukey’s post hoc test: Ancestor versus T600 *P* < 0.0001, T600 versus T600-Hsc82OE *P* < 0.0001). (**C**) Cellular aspect ratio quantification of Ancestor and radicicol-treated Ancestor (*n* = 300 cells, *t* = 16.1, *P* < 0.0001, two-sample *t* test). (**D**) Cluster size determined as cluster radius (in micrometers) for T600 and T600-Hsc82OE (T600 *n* = 1033, T600-Hsc82OE *n* = 3489 from four clones), (*F*_1,6229_ = 3024, *P* < 0.0001, one-way ANOVA, Tukey’s post hoc test: T600 versus Hsc82OE *P* < 0.0001). (**E**) Diagram summarizing the competition assay method in (**F**). Created with Biorender.com. (F) Competition assay measuring the fitness over three rounds of growth and selection, represented as a selection rate constant, for the T600 isolate and four independently generated clones of T600-Hsc82OE competed against T600-GFP (*n* = 4 per competition). To account for the cost of GFP expression, all selection rate constants were normalized by the mean of the T600 versus T600-GFP competition. (*n* = 3 per competition) (*F*_1,14_ = 8.4, *P* = 0.015, nested ANOVA). All values represent means ± SEM.

We next sought to determine whether the reduced Hsp90 expression is a multicellular adaptation, giving rise to novel, beneficial multicellular traits in evolved snowflake yeast. To investigate this, we restored the expression level of Hsp90 back to the ancestral level by integrating *TEF1pr-HSC82* into a safe harbor locus in the snowflake genome (T600-Hsc82OE). This led to a ~50% increase in *HSC82* mRNA expression (fig. S2A). Restoring *HCS82* levels led to cells reverting to shorter and rounder morphology (Fig. 2A), with a significant reduction in mean aspect ratio from 2.93 in wild-type T600 cells to 2.18 in T600-Hsc82OE (Fig. 2B). To further validate that a reduction in Hsp90 expression affects cell shape, we subjected Ancestor cells to short-term treatment with radicicol, a chemical inhibitor of Hsp90. This had a modest but significant effect on the Ancestor, increasing the cellular aspect ratio by ~10% after just 6 hours of growth (Fig. 2C).

We then tested whether the increased aspect ratio created by reduced Hsp90 expression is adaptive under our selective regime (24 hours of growth in shaking incubation, followed by settling selection favoring larger groups). Our previous work has established that cellular elongation is a key trait underpinning the origin of increased multicellular size and toughness, convergently evolving in all five replicate populations that evolved macroscopic size (*18*, *26*). As expected, overexpressing Hsp90 markedly reduced cluster size in four independently generated PA5 T600-Hsc82OE clones, with a mean 3.7-fold reduction in size (Fig. 2D and fig. S2B). We also examined the impact of increased Hsp90 expression on fitness via competition experiments (Fig. 2E). Restoring Hsp90 to ancestral levels was costly, resulting in a mean selection rate constant of −1.57 for the 3 days of competition (Fig. 2F). Together, these results establish that down-regulation of Hsp90 is an adaptive multicellular trait, increasing cellular aspect ratio, and thus multicellular size and fitness.

### Convergent evolution of reduced Hsp90 expression

**Fig. 3.**
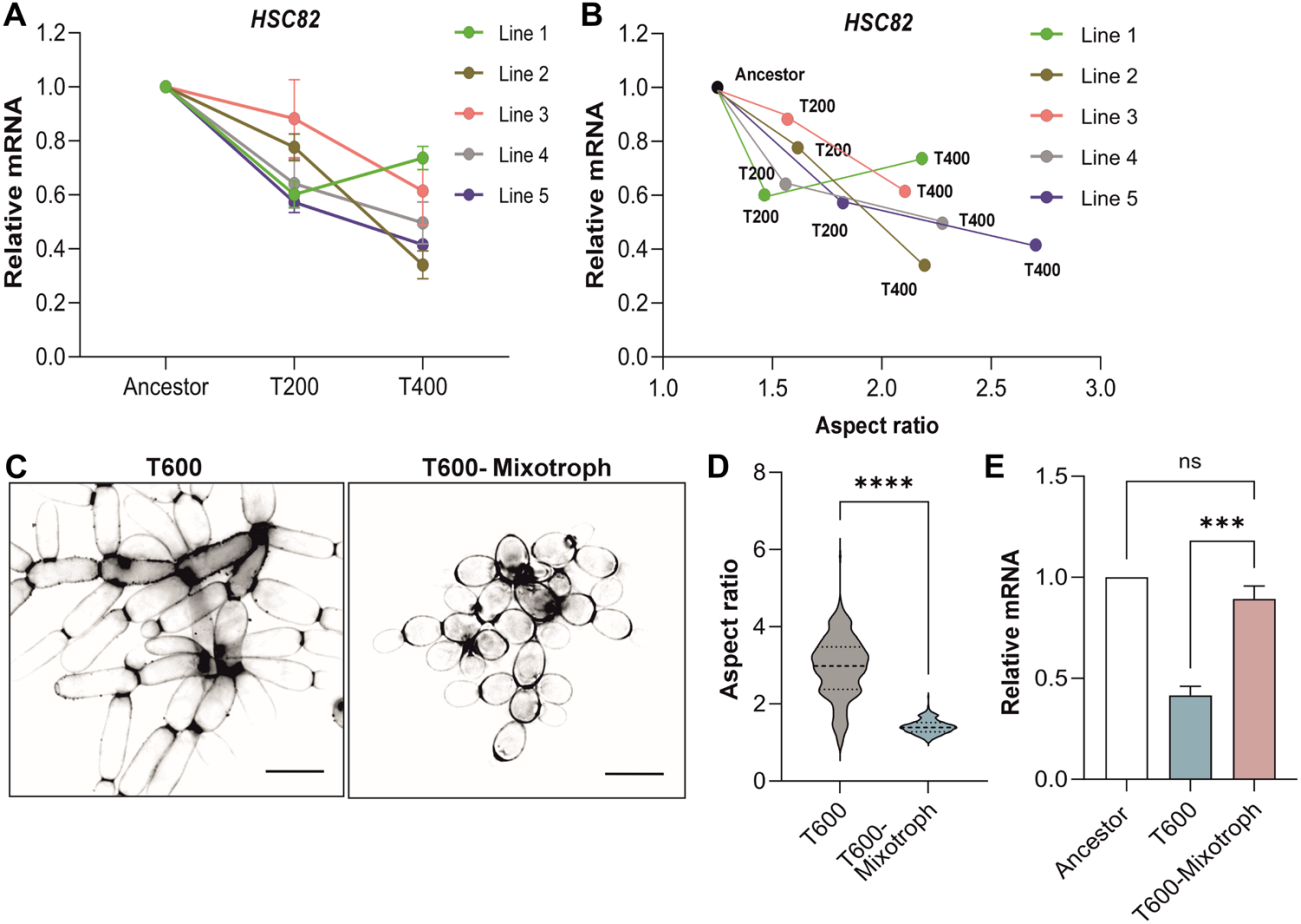
Expression of *HSC82* affects the timing and extent of macroscopic development. (**A**) RT-qPCR quantification of *HSC82* expression in T200 and T400, compared to Ancestor for the five independent lines of anaerobic snowflake yeast (*n* = 4, *F*_*8*__,20_ = 4.485, *P* = 0.003, two-way ANOVA, Tukey’s post hoc test: Ancestor versus T200 *P* < 0.0001, Ancestor versus T400 *P* < 0.0001, T200 versus T400 *P* = 0.025). (**B**) *HSC82* expression against aspect ratio for T200 and T400 of each of the five lines of aerobic snowflake yeast (*r* = 0.74, *P* = 0.009, *y* = −0.33*x* + 1.26, linear regression). (**C**) Representative images of calcofluor-stained T600 and T600-Mixotroph cells to highlight differences in cellular morphology. Scale bars, 10 μm. (**D**) Cellular aspect ratio quantification of T600 and T600-Mixotroph cells (*n* = 300 cells, *t* = 32.58, *P* < 0.0001, two-sample *t* test). (**E**) RT-qPCR quantification of *HSC82* expression in T600 and T600-Mixotroph compared to Ancestor (*n* = 3, *F*_2,6_ = 48.33, *P* = 0.002, one-way ANOVA, Tukey’s post hoc test: Ancestor versus T600-Mixotroph *P* = 0.28, T600-Mixotroph versus T600 *P* < 0.0007). All values represent means ± SEM.

Macroscopic cluster size evolved convergently across the five replicate populations of our anaerobic yeast (PA1–5) through cellular elongation and entanglement (*19*). To determine whether this trait is driven by reduced Hsp90 expression in all replicate populations, we examined *HSC82* expression as they were evolving large size (transfers 0 to 400). All five lines showed significant declines in *HSC82* at T200 and T400, relative to the Ancestor (Fig. 3A). Moreover, changes in the Hsp90 expression level, cellular aspect ratio (Fig. 3B), and cluster size (fig. S3A) are correlated. Replicate lines 2, 4, and 5 show the steepest drop in Hsp90 levels as multicellular evolution progressed. Correspondingly, these populations led to the largest and most rapid change in cellular aspect ratio and cluster size (Fig. 3B and fig. S3A).

Last, we wanted to determine whether reduced Hsp90 expression is simply a by-product of ~3000 generations of evolution and not directly related to the evolution of larger group size. To this end, we measured Hsp90 expression levels in mixotrophic snowflake yeast, a separate treatment of the MuLTEE that was begun with snowflake yeast capable of aerobic metabolism. This yeast underwent the same 600 days of settling selection but did not evolve macroscopic size or elongated cellular morphology (Fig. 3, C and D). Prior work on this system has shown that, under our laboratory conditions, the ability to use oxygen for growth inhibits the evolution of large size. This is because oxygen diffuses poorly through the cluster and is mainly used by surface cells, incurring a size-dependent growth cost that is absent in anaerobic populations (*18*, *19*). In contrast to the macroscopic strains which evolved under anaerobic metabolism, the mixotrophic T600 strain expressed *HSC82* at the same level as the anaerobic Ancestor and displayed approximately 50% higher expression than the anaerobic PA5 T600 strain (Fig. 3E). Collectively, these results provide evidence that down-regulation of Hsp90 has occurred convergently to facilitate the evolution of macroscopic multicellularity.

### Loss of Hsp90 client Cdc28 leads to adaptive morphogenesis of evolved snowflake yeast

**Fig. 4.**
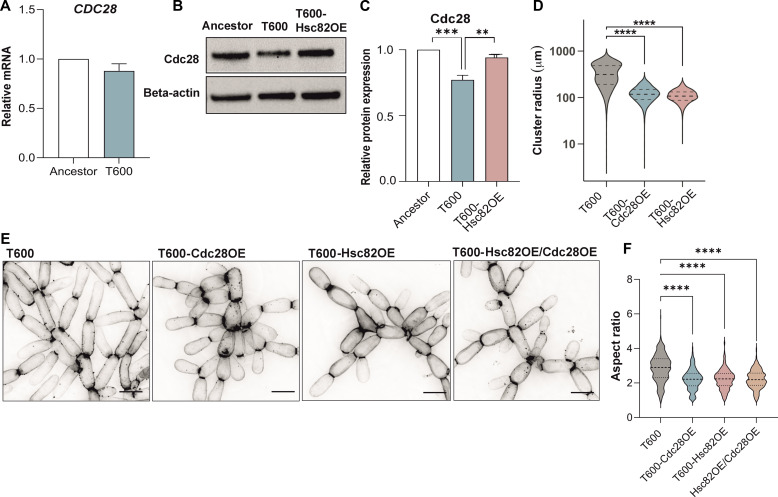
Elongation of T600 cells is driven by Hsc82-mediated destabilization of Cdc28. (**A**) RT-qPCR quantification of *CDC28* expression in T600 compared to Ancestor (*n* = 4, *t* = 1.67, *P* = 0.16, two-sample *t* test). (**B**) Representative immunoblot of Cdc28 in Ancestor, T600, and T600-Hsc82OE with beta-actin as a loading control. (**C**) Quantification of relative band intensity of T600 Cdc28 and T600-Hsc82OE Cdc28 in comparison to Ancestor Cdc28 (*n* = 4, *F*_2,9_ = 22.35, *P* = 0.0003, one-way ANOVA, Tukey’s post hoc test: Ancestor versus T600 *P* = 0.0003, T600 versus T600-Hsc82OE *P* = 0.0025). (**D**) Cluster size determined as cluster radius (in micrometers) for T600, T600-Cdc28OE, and T600-Hsc82OE (T600 *n* = 2742, T600-Cdc28OE *n* = 4458 from four clones, T600-Hsc82OE *n* = 3489 from four clones, *F*_1,7195_ = 3477, *P* < 0.0001, one-way ANOVA, Tukey’s post hoc test: T600 versus T600-Cdc28OE *P* < 0.0001). (**E**) Representative images of calcofluor-stained T600, T600-Cdc28OE, T600-Hsc82OE, and T600-Hsc82OE/Cdc28OE cells to highlight cellular morphology. Scale bars, 10 μm. (**F**) Cellular aspect ratio quantification of T600, T600-Cdc28OE, T600-Hsc82OE, and T600-Hsc82OE/Cdc28OE (*n* = 400 cells, T600-Cdc28OE, and T600-Hsc82OE from four combined clones, T600-Hsc82OE/Cdc28OE from three combined clones; *F*_*3*,1596_ = 25.25, *P* < 0.0001, one-way ANOVA, Tukey’s post hoc test: T600 versus T600-Cdc28OE *P* < 0.0001, T600 versus T600-Hsc82OE *P* < 0.0001, T600 versus T600-Hsc82OE/Cdc28OE *P* < 0.0001).

Cdc28 is the catalytic subunit of the yeast cyclin–dependent kinase that acts as a master regulator of the mitotic cell cycle through multiple combinatorial effects (*27*). Cdc28 is also a folding client of Hsp90 (*28*, *29*). Inhibiting Hsp90 can alter Cdc28 protein expression, which in turn is linked to morphological changes (*28*–*32*). We examined whether Cdc28 is the downstream target of Hsp90 responsible for driving the elongated cell morphology of T600 cells. Our RNA-seq showed no significant difference in *CDC28* mRNA expression between Ancestor and T600 cells, which we confirmed by qPCR (Fig. 4A). However, Cdc28 protein expression was ~25% lower in T600 cells (Fig. 4, B and C). We then investigated whether the reduced stability of Cdc28 protein was caused by reduced Hsp90 expression by examining whether overexpressing Hsc82 increased Cdc28 abundance. We found that Hsc82 overexpression in T600 cells restored ~80% of the reduced Cdc28 protein expression that evolved more than 600 transfers (Fig. 4C), demonstrating that it is a target of Hsp90 that becomes destabilized in the T600 cells due to reduced Hsp90 expression.

Hsp90 has hundreds of client proteins (*33*–*35*). To test whether the down-regulation of Cdc28 is responsible for the cellular elongation phenotype downstream of Hsp90, we inserted a single copy of *CDC28* under its own promoter in a safe harbor locus in the T600 strain (T600-Cdc28OE). This restored the expression of Cdc28 close to that of the Ancestor strain (the average relative expression of T600-Cdc28OE was 1.25 ± 0.14 times that of the Ancestor strain from four clones) (fig. S4, A and B). Notably, this minor elevation in Cdc28 levels resulted in a significantly smaller cluster size, resembling that of T600-Hsc82OE (Fig. 4D and fig. S4C). We then examined whether this effect was due to changes in cellular morphology by measuring the cellular aspect ratio. This showed that the restoration of Cdc28 expression resulted in cells with a smaller, rounder morphology, phenocopying the cellular aspect ratio of *HSC82* overexpressing T600 cells (Fig. 4, E and F). Last, to determine whether Hsc82 and Cdc28 exert their effects on cellular elongation through the same pathway, we conducted an epistasis analysis by simultaneously restoring the expression of both genes. Combined overexpression did not show additive effects on cellular aspect ratio compared to the single overexpression of either gene, demonstrating that Cdc28 is a key downstream factor in Hsp90-mediated control of cellular elongation in T600 cells (Fig. 4F).

**Fig. 5.**
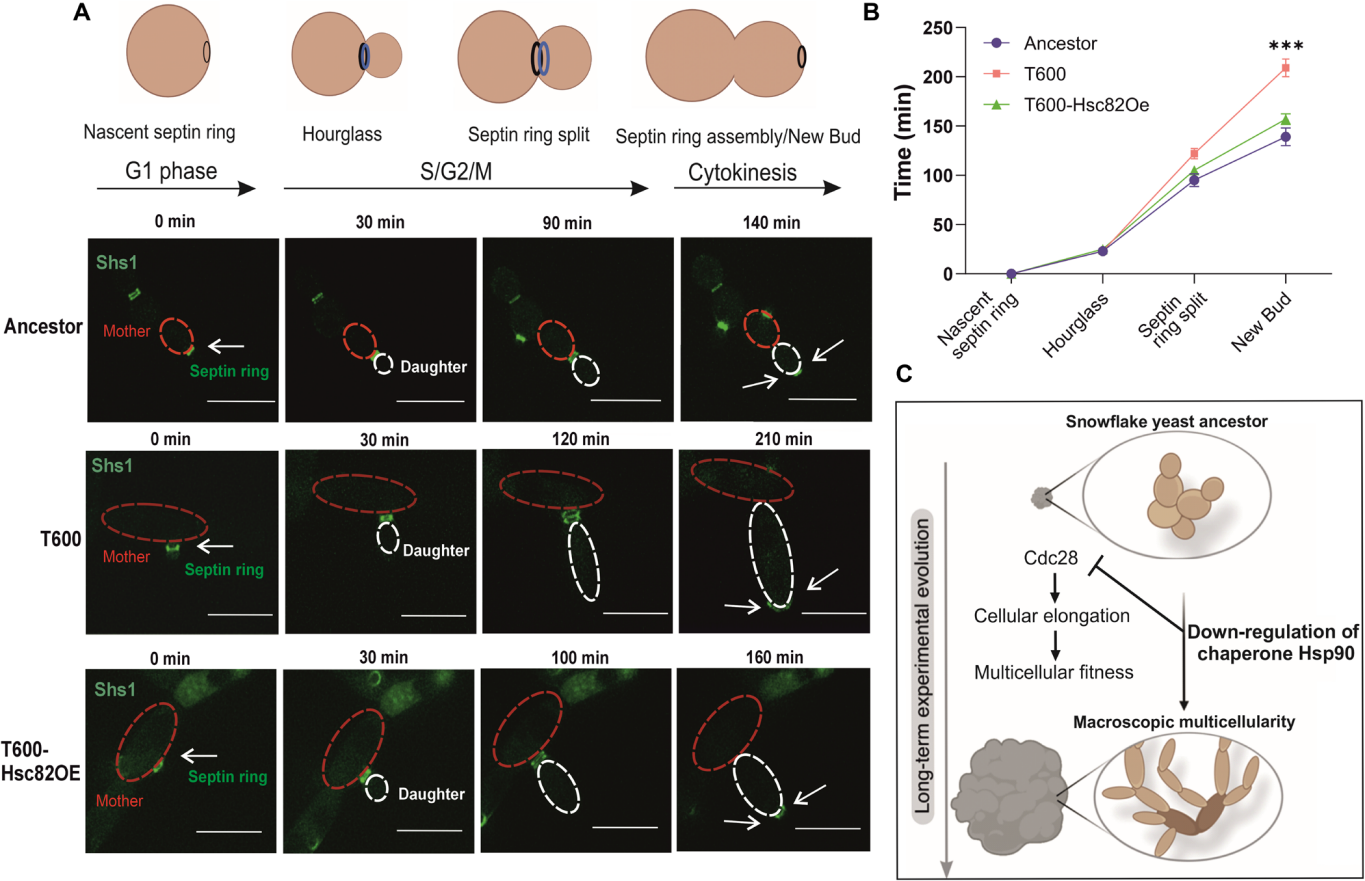
Reinstatement of Hsc82-levels rescue the delayed cell cycle kinetics. (**A**) Representative images of Shs1-mNEONgreen expressed by Ancestor, T600, and T600-Hsc82OE. The mother cell was highlighted with a red dashed line and the daughter cell with a white dashed line. Scale bar, 10 μm. The graphic summarizes the relationship between the septin ring stage and the cell cycle stage. Created with Biorender.com. (**B**) Graphical representation of time-lapse images for Ancestor, T600, and T600-Hsc82OE (*n* = 10, *F*_6,72_ = 14.52, *P* < 0.0001, two-way ANOVA, Tukey’s post hoc test: New Bud Ancestor versus New Bud T600 *P* = 0.0002, New Bud T600 versus New Bud T600-Hsc82OE *P* = 0.0008, New Bud Ancestor versus New Bud T600-Hsc82OE *P* = 0.18). (**C**) Graphical model for how down-regulation of Hsp90 acts on Cdc28 to delay the cell cycle leading to the cellular elongation required for macroscopic evolution during the MuLTEE. Created with Biorender.com. All values represent means ± SEM.

Cell cycle and cell growth are loosely coupled (*36*). We postulated that the Hsp90-mediated reduction of Cdc28 may lead to excessive polarized growth due to delay in mitotic progression, as shown for some *CDC28* mutants (*30*, *37*). To investigate this, we analyzed cell cycle dynamics by using a mNEONgreen-tagged copy of the septin ring subunit Shs1 as a proxy for cell cycle progression (*38*). T600 cells showed delayed mitosis with slower progression of septin ring splitting and late mitotic events relative to Ancestor cells (Fig. 5, A and B). To investigate whether this was due to down-regulated Hsp90, we overexpressed *HSC82* in T600 cells. This accelerated the cell cycle, and T600 cells were now progressing through mitosis at nearly the speed of Ancestor cells, providing evidence that Hsp90-mediated adaptive cellular morphogenesis in T600 is coupled to the progression of the cell cycle. These results provide evidence that adaptive multicellular morphogenesis established by the Hsp90-Cdc28-axis acts by delaying the cell cycle kinetics, enabling cells to undergo prolonged polarized growth that leads to their elongation (Fig. 5C).

## DISCUSSION

In this work, we investigated the molecular basis of multicellular adaptation during the MuLTEE. We found that down-regulation of the chaperone protein Hsp90 was a convergent and adaptive trait that drove cellular elongation by modulating the stability and activity of the central cell cycle kinase Cdc28. This delayed progression through the cell cycle results in prolonged polarized growth and the formation of elongated cells, which generate biomechanically tough multicellular groups with higher Darwinian fitness under our selection scheme. Our results thus reveal how manipulation of ancient systems that guide protein folding can facilitate major evolutionary transitions by generating novel developmental phenotypes through proteostatic tuning.

Previous research has established that Hsp90 can influence phenotypic variation and evolvability in a broad range of multicellular organisms by buffering or revealing cryptic genetic variation (*39*–*44*). Most prior work has examined how environmental stress acts as a catalyst for changes in Hsp90 function, subsequently leading to the appearance of Hsp90-dependent phenotypic alterations. In contrast, we demonstrate that Hsp90 can be under long-term selection for its role in regulating gene expression, generating novel, adaptive traits by modifying the activity of key client proteins. Our results are in line with a recent study showing that the introduction of a heterologous copy of Hsp90 from evolutionarily divergent *Yarrowia lipolytica* to *S. cerevisiae* broadened the phenotypic space for natural selection (*45*). Together, these studies establish that the Hsp90 function can be evolutionarily tuned to facilitate rapid adaptation.

At first glance, the loss of Hsp90 activity appears to contradict the broader macroevolutionary trend, in which increasingly complex organisms evolve increasingly complex proteomes (*46*). However, this largely occurs through an increase in the number of co-chaperones, rather than core chaperones, some of which have actually been lost before major evolutionary transitions. Comparative analysis of genes lost in animals revealed that Hsp100/ClpB chaperones are present in the closest living relatives of animals but are lost at the base of metazoa (*46*, *47*). Such loss of core chaperones before the rapid diversification of animals might indicate an adaptive role for altered proteostatic tuning during the early stages of multicellular evolution.

Despite their key housekeeping functions, chaperone expression is readily tunable. For instance, different animal tissues display distinctive chaperone expression profiles that specify their protein folding capacity (*48*–*50*). In yeast, Hsp90 chaperoning capacity surpasses demand under normal conditions as cells are viable with as little as 5% of wild-type Hsp90 under optimal growth conditions (*23*). Given the possibility for plasticity in Hsp90 expression, one possible alternative explanation for our results is that Hsp90 down-regulation is not directly linked to multicellular adaptation; instead, it might be a by-product of metabolic changes or other mutations that occurred during the MuLTEE. This is unlikely; however, given that Hsp90 down-regulation was observed in all five replicate populations that evolved macroscopic size, restoring Hsp90 levels to the ancestral level reversed the cellular elongation phenotype and reduced multicellular fitness, and Hsp90 expression did not decrease in a parallel evolutionary treatment which remained microscopic. Instead, our data indicate that the down-regulation of Hsp90 derives from decreased transcriptional activity of Hsf1. Although Hsf1 expression was unaltered, its activity can be modulated by posttranslational modifications that affect its DNA binding without affecting its expression (*51*–*53*), which is consistent with what was observed in the T600 cells. Last, while we provide evidence that the cellular elongation phenotype driven by reduced Hsp90 is mediated through its client Cdc28, we also note that Hsp90 has hundreds of other clients. It is therefore plausible that altered chaperoning of other proteins may contribute to the emergence of additional multicellular phenotypes.

Our results have several implications for the continued evolution of multicellularity in the MuLTEE. First, the evolution of increased cell aspect ratio via cell cycle delay may help entrench multicellularity. Entrenchment is a process through which multicellular traits become stabilized over evolutionary time, reducing the likelihood of reversion to unicellularity (*54*). We show here the reduction of Hsp90 promotes multicellular fitness by mediating cellular elongation via a delayed cell cycle. However, at the single-cell level, delaying progression through the cell cycle should be costly, as unicellular fitness in laboratory populations is primarily dependent on a genotype’s growth rate (*55*). Second, increased cellular aspect ratio was not driven exclusively by Hsp90—macroscopic lineages had an average of 32 mutations by T600, many of which are in genes affecting the cell cycle, filamentous growth, and budding processes (*19*). Mutations in some of these genes increase cellular aspect ratio and group size independently from Hsp90 (*19*, *56*), and future work will be required to explore the joint evolution of genetic and epigenetic mechanisms underpinning the origin and maintenance of novel multicellular traits. Last, it will be interesting to further examine the permanence of the altered heat shock response, Hsp90 expression, and their consequences on protein evolution and stress tolerance over long evolutionary time scales (*57*).

Overall, our study shows how Hsp90, a key protein chaperone in all eukaryotes, influences the evolution of multicellularity in *S. cerevisiae*. By modulating the proteostasis of its client proteins, Hsp90 can generate multicellular phenotypes that are adaptive and heritable. This reveals how protein folding systems can shape emerging multicellular genotype-phenotype relationships, supporting the progression of an evolutionary transition in individuality. Our approach, using long-term open-ended experimental evolution, opens possibilities for fundamental biological discovery, and highlights the importance of epigenetic mechanisms in the transition to multicellularity.

## MATERIALS AND METHODS

### Methods

#### Snowflake yeast strains and long-term evolution experiment

All yeast strains are derived from the Y55 diploid background. The ancestor strain was generated by deletion of both copies of the *ACE2* transcription factor (*ace2*Δ*::KanMX/ ace2*Δ *:: KanMX*) and selecting a randomly generated petite mutant (i.e., carrying deletions in its mitochondrial DNA that render it nonfunctional). This anaerobic yeast snowflake strain was further subjected to a long-term evolution experiment (*19*). Briefly, five replicate populations of Ancestor snowflake were grown in 10 ml of YPD media (1% yeast extract, 2% peptone, and 2% dextrose) for 24 hours, using settling selection to select for larger cluster size. Every 24 hours over 600 consecutive days, 1.5 ml of culture was transferred into 1.5-ml Eppendorf tubes and left on the bench for 3 min to allow cell settling [time for settling reduced once macroscopic size evolved, see (*19*) for details]. The bottom 50 μl of the settled culture was then transferred into fresh 10 ml of YPD for the next round of 24-hour growth and settling selection.

### Experimental conditions and treatments

Before experiments, cells were grown in a YPD medium (1% yeast extract, 2% Bacto peptone, and 2% glucose) for 24 hours and subject to daily settling selection. In total, three rounds of growth and settling selection were applied. On the fourth day, cells were subject to settling selection, grown for 4 to 6 hours to exponential phase then experiments were conducted. For heat shock experiments, cells were incubated at 42°C for 30 min, and then allowed to recover at 30°C for 10 min and RT for 5 min. For treatment of Ancestor cells with radicicol, cells were incubated at 30°C with a final concentration of 40 μm of radicicol solubilized in dimethyl sulfoxide (DMSO) for 6 hours. Control cells were incubated with an equivalent volume of DMSO.

### Transformation of snowflake yeast

Ancestor, T200, and T400 snowflake yeast strains were transformed via a standard lithium acetate (LiAc) method (*58*). Cells were grown in the exponential phase in a YPD medium (1% yeast extract, 2% Bacto peptone, and 2% glucose) for 24 hours. After 24 hours, 250 μl of biomass (per transformation) was transferred to 10 ml of YPD and grown for 4 hours. Cells were then pelleted via centrifugation at 1000*g* for 1 min and washed once with H_2_O and once with 10 mM LiAc. Cells were pelleted and resuspended in 240 μl of polyethylene glycol (PEG) buffer (40% PEG-3350 (m/V), and mixed with 36 μl of 1 M LiAc, plasmid DNA (1 μg) or PCR product (10 μl), and 10 μl of salmon sperm DNA (100 mg/ml) (before use, boiled for 10 min at 100°C and cooled down on ice). Yeast was then subjected to heat shock at 42°C for 30 min and spun down at 300*g* for 5 min. Cells were incubated in 3 ml of YPD media for 3 hours and then plated on YPD plates with appropriate antibiotics. Snowflake yeast strain T600 was transformed via electroporation. Cells were grown in a YPD medium (1% yeast extract, 2% Bacto peptone, and 2% glucose) for 24 hours. After 24 hours, 100 μl of biomass (per transformation) was transferred to 10 ml of YPD and grown for 4 hours. Cells were then pelleted by centrifugation at 1000*g* for 5 min and washed twice with H_2_O and once with 1 M sorbitol. Cells were incubated in 10 ml of conditioning solution (0.1 M LiAc/10 mM dithiothreitol) at 30°C for 30 min. A cell pellet was collected by centrifugation at 1000*g* for 5 min, and then washed twice with 1 M sorbitol. Cells were resuspended in 350 μl of electroporation buffer (1 M sorbitol/1 mM CaCl_2_) and mixed with plasmid DNA (1 μg) or PCR product (10 μl). The transformation mixture was transferred to electroporation cuvettes (Bio-Rad), and electroporation was conducted at 2.5 kV (Bio-Rad micropulser). Cells were transferred to 5 ml of YPD to recover overnight, and then plated on plated on YPD plates with appropriate antibiotics.

### Protein extraction and Western blot

Cells were harvested by centrifugation at 1000*g* for 5 min and the pellet was washed once with H_2_O and once with phosphate-buffered saline (PBS). Cells were resuspended in 800 μl of PBS and 200 μl of 50% trichloroacetic acid (TCA) and stored for 30 min at −80°C to allow protein precipitation. TCA-treated cells were pelleted, washed twice with ice-cold 80% acetone, and air-dried. Pellets were dissolved in 180 μl of lysis buffer (1% SDS and 0.1 M sodium hydroxide) and boiled for 5 min. Protease inhibitors were then added (Roche). Quantification of protein concentration was done using a BCA Protein Assay Kit (Thermo Fisher Scientific). For Western blot, 5 to 50 μg of protein was mixed with 6× loading buffer (48% glycerol and 0.03% bromophenol blue) and run through SDS-PAGE gel. For detection, primary mouse Anti-Hsp90 (StressMarq) was used 1:1000; primary mouse Anti-Cdc28 (Santa Cruz Biotechnology Inc) was used 1:1000; and for loading control, mouse Anti–β-actin (Abcam) was used 1:1000. For secondary antibody, horseradish peroxidase–conjugated Rabbit Anti-Mouse (Invitrogen) was used 1:2000. Relative band density was analyzed using FIJI ImageJ software. Bands were normalized to beta-actin loading control.

### RNA extraction and RT-qPCR

Cells were harvested by centrifugation at 1000*g* for 5 min. Cell pellets were resuspended in 800 μl of TRI Reagent (Zymo Research), and then transferred to 2-ml Touch Micro-Organism Lysing Mix tubes (Omni). Cells were then mechanically disrupted with Precellys 24 Tissue Homogenizer (Bertin Instruments) at 6000 rpm for 15 s for a total of four cycles with a 2-min break on ice between cycles. RNA was purified using a Direct-zol RNA MiniPrep Kit (Zymo Research) and following the manufacturer’s instructions. RNA concentration and purity were measured by NanoDrop, and RNA samples were stored at −80°C. cDNA was prepared from purified RNA (1 μg) using Oligo(dT) (Invitrogen) and reverse transcriptase SuperScript IV (Invitrogen) according to the manufacturer’s instructions. Synthesized cDNA was diluted 1:10 and 5 μl mixed with SsoAdvanced Universal Sybr Green Supermix (Bio-Rad). qPCR was performed using the Bio-Rad CFX96 PCR machine. Relative RNA levels were quantified using the ΔΔCT method. *ACT1* was used as the normalization control.

### RNA sequencing and data analysis

RNA for sequencing was extracted as described above. RNA-seq library preparation and sequencing was performed by the Sequencing Unit at the Institute of Molecular Medicine Finland (FIMM) Technology Centre, University of Helsinki. Illumina TruSeq Stranded mRNA Library Preparation Kit (Illumina) and NextSeq 500 Mid Output Kit PE75 (120 M reads) (Illumina) were used following the manufacturer’s instructions. Quality control of raw reads was performed with FastQC v0.11.9, followed by read filtering with Trim Galore v0.6.6 and ribosomal RNA removal with SortMeRNA v4.2.0. Transcriptome mapping was done with Salmon v1.4.0, and quality control of read mapping was done with STAR v2.7.8a and Qualimap v2.2.2d. Summary of quality control results was reported with MultiQC v1.9. Differentially expressed genes between Ancestor and T600 cells were found using DESeq2 v1.32.0 (adjusted *P* < 0.05, Wald test) in R 4.1.0. Functional enrichment of differentially expressed genes by overrepresentation analysis was done with clusterProfiler v4.0.0. For this study, the yeast genome/transcriptome references and gene annotations from Ensembl release 103 were used, which are based on yeast S288C genome assembly R64-1-1.

### Hsf1 luciferase activity assay

Snowflake yeast strains were transformed with genomically integratable linearized plasmid, pCYB2194-3xHSE-NLucPEST-HygMX, which is based on the plasmid pAM17 (P_cyc1-3xHSE-_yNlucPEST) (a generous gift from C. Andréasson/Stockholm University). Cells were grown in standard experimental conditions and induction of Hsf1 was done with heat shock at 42°C for 10 min. Cells were washed once with H_2_O and then mixed with 400 μl of lysis buffer [50 mM tris-HCl (pH 7.5), 300 mM NaCl, and 10 mM imidazole]. Proteins were extracted by mechanical disruption with Precellys 24 Tissue Homogenizer (Bertin Instruments) at 6000 rpm for 15 s for a total of four cycles with a 2-min break on ice between cycles. Protein concentration was measured using a NanoDrop (Thermo Fisher Scientific) and a BCA Protein Assay Kit (Thermo Fisher Scientific). Nano-Glo substrate (Promega GmbH, Germany) was prepared at 1:100 substrate to buffer and mixed 1:10 (20 μl) with lysate (180 μl at a concentration of 0.5 μg/ml) in a 96-well plate. Bioluminescence was measured immediately, using an Enspire Plate Reader (PerkinElmer).

### Hsf1 ChIP-qPCR assay

Ancestor and T600 cells, where endogenous Hsf1 was C-terminally tagged with a green fluorescent protein (GFP), were grown under standard experimental conditions. Cells were then treated with formaldehyde (1% final concentration) for 15 min at RT for protein cross-linking. The cross-linking reaction was quenched by adding glycine (final concentration, 125 mM) and incubating for 5 min. Cells were collected by centrifugation at 1000*g* for 5 min at 4°C. The supernatant was removed, the pellet was resuspended in 10 ml of TBS (pH 7.5), and then centrifuged again at 1000*g* for 5 min at 4°C. Cells were washed with ice-cold H_2_O, pelleted, and resuspended in 800 μl of lysis buffer [50 ml of 1 M Hepes/KOH (pH 7.5), 28 ml of 5 M NaCl, 2 ml of 500 mM EDTA, 100 ml of 10% Triton X-100, 1 g of Na-deoxycholate, cOmplete Protease Inhibitor Cocktail tablet (Roche), and 1 mM phenylmethylsulfonyl fluoride]. Cells were lysed with the Precellys 24 Tissue Homogenizer (Bertin Instruments) at 6000 rpm for 15 s for a total of four cycles with a 2-min break on ice between cycles. Lysates were recovered and subjected to sonication using a Bandolin Sonopuls sonicator (10 cycles with 30-s pulse). Cell debris was removed by centrifugation at 3000 rpm for 5 min at 4°C. To perform immunoprecipitation, the equivalent of 500 to 800 μg of protein was incubated with 1 μg of anti-GFP antibody (Sigma-Aldrich) and then incubated with Protein A/G magnetic beads (Thermo Fisher Scientific) overnight at 4°C. Beads were washed with lysis buffer and eluted with TE/1% SDS (pH 8.0). Cross-links were reversed by incubation at 65°C, and elutants were treated with ribonuclease A and proteinase K. DNA was purified using a GeneJET PCR purification kit (Invitrogen). Immunoprecipitated DNA was mixed with SsoAdvanced Universal Sybr Green Supermix (Bio-Rad) and qPCR was conducted using the Bio-Rad CFX96 PCR machine. Primers against the HSE regions of *HSC82* and *HSP82* were used. DNA levels were quantified as a percentage of input. The background was determined by signal arising from incubating an equivalent volume of chromatin extract without antibody. Signal from input was used to normalize against variation in yield of chromatin.

### Microscopy

For still images, cells were stained with calcofluor (100 μg/ml) for 5 min. Cells were washed twice with H_2_O, and then resuspended into 1 ml of H_2_O. Ten microliters of cell suspension was dropped onto a slide and a coverslip was firmly placed over the top. For time-lapse experiments, cells were grown in YPD for 24 hours, and then transferred to synthetic complete (SC) media and grown for 2 hours. Clusters were then broken via mechanical disruption between two glass slides and transferred to a 96-well thin glass-bottom imaging plate precoated with concanavalin A (Sigma-Aldrich) in a volume of 200 μl of SC media. Well coating was performed by adding 100 μl of concanavalin A (2 mg/ml in PBS), incubating at room temperature for 30 min, and washing wells twice with PBS. Imaging was performed with a customized Olympus IX-73 inverted widefield fluorescence light microscope DeltaVision Ultra (GE Healthcare) equipped with Pco edge 4.2ge sCMOS camera and CentOS 7 Linux operating system. Images were taken at 60× oil objectives and, depending on the fluorophore properties, Blue (excitation, 390 nm; emission, 435 nm) and Green (excitation, 475 nm; emission, 525 nm) filter settings. Imaging data were analyzed with FIJI ImageJ software.

### Competition assay

We measured the fitness of Hsc82 overexpression strains over three transfers of growth and settling selection. To hedge against possible off-target effects of genomic transformation, we replicated this experiment four times, with four independently generated Hsc82OE clones. In each comparison, we competed four replicate populations of T600-GFP against an unmarked Hsc82OE strain. Before the competition, we inoculated each strain into 10 ml of YPD and grew them for 5 days of growth and settling selection to allow each population to reach cluster size equilibrium. Each strain was then mixed in equal volumes with T600-GFP, and 50 μl of this mixture was inoculated into 10 ml of YPD media to start the competition. Every 24 hours for 3 days, 1 ml of each competing population was transferred into 1-ml centrifuge tubes to perform settling selection. After 3 min of settling on the bench, the top 950 μl was discarded. The remaining 50 μl was transferred into 10 ml of fresh YPD media. The cellular biomass of GFP-tagged and non–GFP-tagged strains were estimated on the basis of the cluster area with the following equation

$$\text{Biomass}=\frac{4}{3}*\pi *{\left(\sqrt{\frac{\text{Area}}{\pi }}\right)}^{3}$$

We used the biomass of GFP-tagged and non–GFP-tagged strains of initial mixtures and 3-day cultures to calculate fitness as a selection rate constant, using the method described by Richard Lenski here: https://lenski.mmg.msu.edu/ecoli/srvsrf.html. To account for the costs of GFP expression, we normalized the selection rate constant of T600-Hsc82OE clones by the selection rate constant of T600 versus T600-GFP (all fitness values shown in Fig. 2F are normalized in this way).

### Statistical analysis

The statistical analysis tests used in this study were the two-tailed Student’s *t* test, one-way analysis of variance (ANOVA), two-way ANOVA, and a nested ANOVA. The analysis was conducted using the Prism statistical software program (version 9.0; GraphPad Software, Boston, MA, USA). A *P* value less than 0.05 was considered statistically significant. In the figure legends, *, **, ***, and **** indicate *P* values less than 0.05, 0.01, 0.001, and 0.0001, respectively. All experiments were performed with three replicates unless specified, and the error bars in the figure legends represent means ± SEM.

### Materials

All materials and strains used in this study can be found in tables S1 to S4.
