# Supplementary material for: Proteostatic tuning underpins the evolution of novel multicellular traits
Source: Sci Adv. 2024 Mar 8;10(10):eadn2706. doi: 10.1126/sciadv.adn2706 (PMC10923498; doi:10.1126/sciadv.adn2706)
Supplement: Supplementary file 1 — Figs. S1 to S4 Tables S1 to S4 [file sciadv.adn2706_sm.pdf]

Science Advances
AAAS

Supplementary Materials for

Proteostatic tuning underpins the evolution of novel multicellular traits

Kristopher Montrose *et al.*

Corresponding author: Juha Saarikangas, juha.saarikangas@helsinki.fi;
William C. Ratcliff, william.ratcliff@biology.gatech.edu

Sci. Adv. **10**, eadn2706 (2024)
DOI: 10.1126/sciadv.adn2706

This PDF file includes:

Figs. S1 to S4
Tables S1 to S4

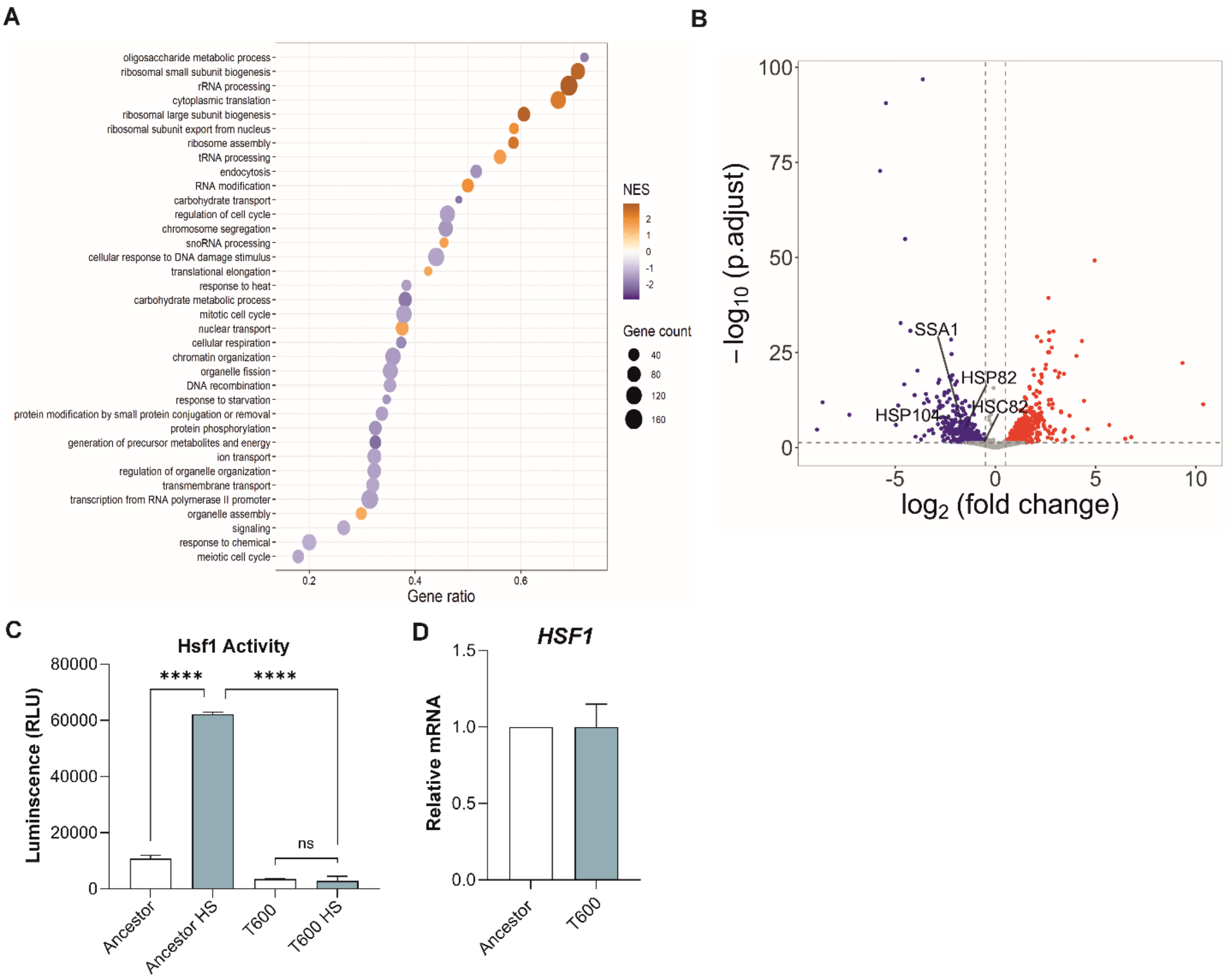

Fig. S1. Hsp90 is downregulated in macroscopic multicellular yeast.
(**A**) Dot plot of differential gene expression between Ancestor and T600 snowflake yeast organized into the top 50 gene ontology hits (*n*=3). (**B**) Volcano plot of differential gene expression between Ancestor and T600 highlighting changes in chaperone proteins Hsp90 (Hsc82 and Hsp82), Hsp70 (Ssa1) and Hsp104 (*n*=3). (**C**) Hsf1 activity before and after heat shock in Ancestor and T600 cells measured as luminescence. Luminescent readings of substrate alone were subtracted as background (n=4, $F_{3, 12}$ = 686.1, $p < 0.0001$, one way ANOVA, *Tukey's* post hoc test Ancestor vs Ancestor HS $p < 0.0001$, T600 vs T600 HS $p = 0.97$, Ancestor HS vs T600 HS $p < 0.0001$). (**D**) Quantification of *HSF1* expression level by RT-qPCR of T600 compared to Ancestor (n=6, t = 0.0071, $p = 0.994$, two-sample t-test). All values represent mean ± SEM.

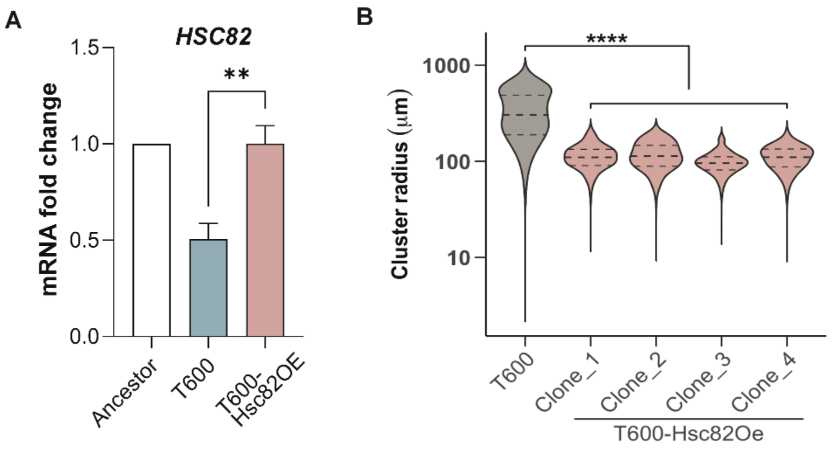

Fig. S2. Overexpression of Hsc82 reduces T600 cluster size.
(**A**) Quantification of *HSC82* expression level by RT-qPCR in T600 and T600-Hsc82OE compared to Ancestor (n=4, $F_{2,6}$ = 15.96, p = 0.004, one way ANOVA, *Tukey's* post hoc test T600 vs T600-Hsc82OE p = 0.006). (**B**) Cluster size as a measure of cluster radius (μm) for T600 and four clones of T600-Hsc82OE ($F_{1,6226}$ = 3024, p < 0.0001, one way ANOVA, *Tukey's* post hoc test T600 vs T600-Hsc82OE 1-4 p < 0.0001). All values represent mean ± SEM.

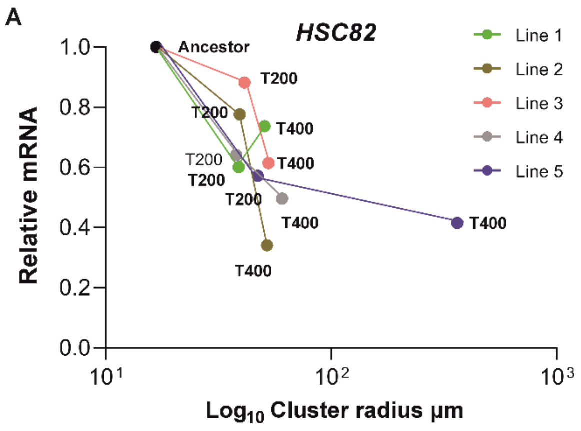

Fig. S3.: ***HSC82* expression correlates with snowflake yeast cluster size.**
(**A**) Scatter plot of *HSC82* expression against cluster radius for T200 and T400 cells for each of the five lines of aerobic snowflake yeast. (r = 0.66, p = 0.02, y = -0.40x+1.33, Linear regression).

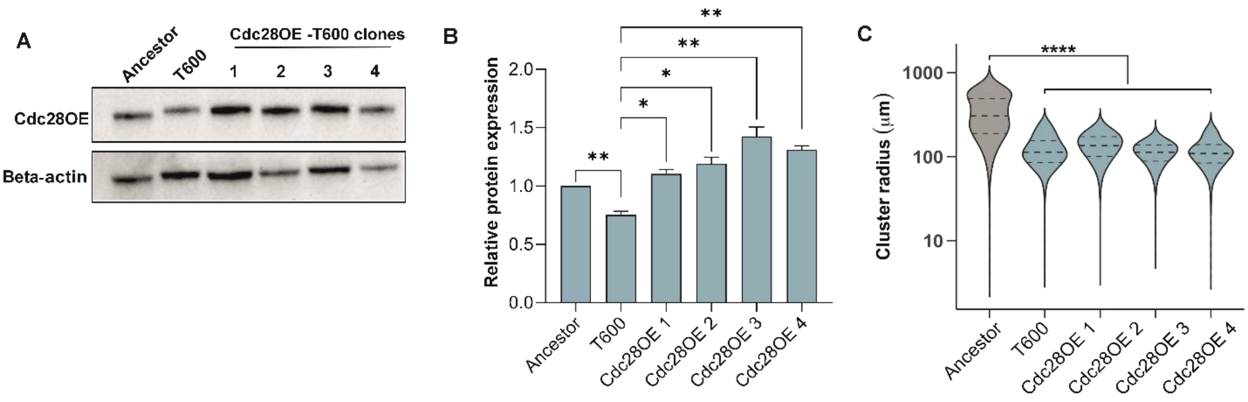

Fig. S4. Overexpression of Cdc28 reduces T600 cluster size.
(**A**) Representative immunoblot of Cdc28 expressed by Ancestor, T600 and 4 clones of T600-Cdc28OE, detected with anti-Cdc28 antibody. Antibody against Beta-actin was used as a loading control. (**B**) Quantification of the band intensity of Cdc28 in T600 and four clones of T600-Cdc28OE relative to the Ancestor. Bands were normalized to loading control (n=5, $F_{2,10} = 27.37$, $p < 0.0001$, one way ANOVA, *Tukey's* post hoc test Ancestor vs T600 $p = 0.0081$, T600 vs T600-Cdc28OE 1 $p = 0.0251$, T600 vs T600-Cdc28OE 2 $p = 0.0116$, T600 vs T600-Cdc28OE 3 $p = 0.006$, T600 vs T600-Cdc28OE 4 $p = 0.002$). (**C**) Cluster size as a measure of cluster radius (μM) for T600 and four clones of T600-Cdc28OE ($F_{1,7195} = 3477$, $p < 0.0001$, one way ANOVA, *Tukey's* post hoc test T600 vs T600-Cdc28OE 1-4 $p < 0.0001$).

Table S1. Antibodies and reagents used in this study

REAGENT or RESOURCE	SOURCE	IDENTIFIER
**Antibodies**		
Mouse Anti-β-actin [8F10-G10]	Abcam	ab1700325<br>RRID:AB_2893492
Mouse Anti-Hsp90	StressMarq Biosciences	SMC-135<br>RRID:AB_2121063
Mouse Anti-Cdc28	Santa Cruz Biotechnology, Inc.	sc-515762
Mouse Anti-GFP	Merck	11 814 460 001<br>RRID:AB_390913
Rabbit Anti-Mouse HRP	Invitrogen	A16166<br>RRID:AB_2534837
**Chemicals, Reagents**		
Concanavalin A	Sigma-Aldrich	Cat # C2010
Radicicol	Sigma-Aldrich	Cat # R2146
100 mg/ml salmon sperm DNA	Sigma-Aldrich	Cat #C2759
Complete™, Mini, EDTA-free Protease Inhibitor Cocktail Tablets	Roche	Cat# 11836170001
Superscript IV reverse transcriptase	Thermo Fisher Scientific	Cat# 18090010
Sso Advanced Universal Sybr Green Supermix	Biorad	Cat# 1725270
Pierce™ Protein A/G Magnetic Beads	Thermo Fisher Scientific	Cat# 88802

Commercial assays		
Easy Clone 2.0 Yeast ToolKit	Addgene	The EasyClone 2.0 kit was a gift from Irina Borodina (Addgene kit # 1000000073)
Pierce™ BCA Protein Assay Kit	Thermo Fisher Scientific	Cat #23227
Direct-zol RNA Miniprep Kit	Zymo Research	Cat #23225
Nano-Glo® Luciferase Assay System	Promega	Cat# N1110
GeneJET PCR purification kit	Thermo Fisher Scientific	Cat# K0701

Table S2. **Strains used in this study**

**Strain Name**	**Genotype**	**Strain ID**	**Reference**
Ancestor	*ace2*::/*ace2*::KanMX	KM2	(*18*)
T200	*ace2*::KanMX/*ace2*::KanMX - 200 days evolved	KM30	(*18*)
T400	*ace2*::KanMX/*ace2*::KanMX - 400 days evolved	KM31	(*18*)
T600	*ace2*::KanMX/*ace2*::KanMX - 600 days evolved	KM34	(*18*)
T600-Mixotroph	*ace2*::KanMX/*ace2*::KanMX - 600 days evolved	KM155	(*18*)
T600-Hsc82OE	*ace2*::KanMX/*ace2*::KanMX *TEF1pr-HSC82*:NatMX	KM175-177	This study
Ancestor Hsf1-GFP	*ace2*::KanMX/*ace2*::KanMX *HSF1-GFP*:HygMX	KM112	This study
T600 Hsf1-GFP	*ace2*::KanMX/*ace2*::KanMX *HSF1-GFP*:HygMX	KM113	This study
T600-Cdc28OE	*ace2*::KanMX/*ace2*::KanMX *CDC28pr-CDC28*:HygMX	KM184-188	This study
Ancestor Shs1-mNEONgreen	*ace2*::KanMX/*ace2*::KanMX *SHS1-mNEONgreen*:NatMX	KM178	This study
T600 Shs1-mNEONgreen	*ace2*::KanMX/*ace2*::KanMX *SHS1-mNEONgreen*:NatMX	KM179	This study
T600 Shs1-mNEONgreen/Hsc82OE	*ace2*::KanMX/*ace2*::KanMX *SHS1-mNEONgreen*:HygMX:: *TEF1pr-HSC82*:NatMX	KM180	This study
T600-Cdc28OE/Hsc82OE	*ace2*::KanMX/*ace2*::KanMX *CDC28pr-CDC28*:HygMX:: *TEF1pr-HSC82*:NatMX	KM189-192	This study

Table S3. **Plasmids used in this study**

Plasmids	Plasmid ID	Reference
pYM25-*yeGF*P-hphNT1	PKM11	Euroscarf
Pam17-*3xHSE*-NLucPEST	PKM19	(*25*)
pCYB2194-*3xHSE*-NLucPEST-HygMX	PKM21	This study
pRS40HO-N-*TEF1pr-HSC82*-NatMX	PKM30	This study
pCYB2194-*Cdc28pr-CDC28*-HygMX	PKM34	This study
pYM1946-*mNEONgreen*–NatMX	PJS108	This study
pCYB2194-*mNEONgreen*–HygMX	PKM33	This study

Table S4. **Primers used in this study**

**Tagging Primers**	**Sequence**
HSF1 F	TACAACGATCACCGCCTGCCCAAACGAGCTAAGAACGTACGCTGCAGGTCGAC
HSF1 R	ACGCTATTTAATGACCTTGCCCTGTGTACTAATCGATGAATTCGAGCTCG
SHS1 F	CACGTATACTGATTTAGCCTCTATTGCATCGGGTAGAGATGGTGACGGTGCTGGTTTA
SHS1 R	TATTTATTTATTTATTTGCTCAGCTTTGGATTTTGTACAGATACAACTCACACAGGAAACAGCTATGACC
**QPCR primers**	
HSC82 F	GAGAGTTGATGAGGGTGGT
HSC82R	GTTAGTCAAATCTTTGACGGT
HSP82 F	GAGTTGACGAAGGTGGTGCT
HSP82 R	ATGCAAAGGAAGTTGGTTCG

CDC28 F	GCCAAGCTTTCCTCAATGGC
CDC28 R	GGGTCATACGCGAGGAGTTT
ACT1 F	ATTATATGTTTAGAGGTTGCTGCTTTGG
ACT1 R	CAATTCGTTGTAGAAGGTATGATGCC
HSF1 F	ATAATGACACCGAGCACGCA
HSF1 R	CATCTACCGTGAGGAAGGGC
**ChIP Primers**	
HSC82 UAS F	CGCCTTTCTGTTTTCTGGGC
HSC82 UAS R	TCACTTACGGTGGGCAGTTC
HSP82 UAS F	CAGTAATCCATAAACCAGTT
HSP82 UAS R	ACAGATGTTAAGAATTGAAGG
